# Implementation of Emotional Connection Training in Pediatric Primary Care: Mixed Methods Study

**DOI:** 10.2196/81250

**Published:** 2026-06-16

**Authors:** Elizabeth Erickson, J Blakely Amati, Dani Dumitriu, Anna Miller-Fitzwater, Debra L Best, Doreet Preiss, Elena Uejo, Adrian Brown, Jennifer E Crotty, Leah Gillen, Cherece Grier, Kristina K Gustafson, Nicole Shearman, Andréane Lavallée, Jennifer Warmingham, Elena Arduin, Nikki Shearman, Jackie Young, Jessica Sperling

**Affiliations:** 1 Department of Pediatrics, Medical Center, Duke University, Durham, NC, United States; 2 Department of Pediatrics, Prisma Health, Greenville, SC, United States; 3 Department of Pediatrics, Center for Early Relational Health, Columbia University Irving Medical Center, 3 Columbus Circle, 11th floor, New York, NY, 10019, United States; 4 Department of Pediatrics, School of Medicine, Wake Forest University, Winston-Salem, NC, United States; 5 Social Science Research Institute, Duke University, Durham, NC, United States; 6 Department of Pediatrics, Brody School of Medicine, East Carolina University, Greenville, NC, United States; 7 Reach Out and Read, Boston, MA, United States; 8 Department of Pediatrics, Medical University of South Carolina, Charleston, SC, United States

**Keywords:** medical education, community pediatrics, developmental-behavioral health, early relational health, resident education

## Abstract

**Background:**

In 2021, the American Academy of Pediatrics released a policy statement spotlighting the health-promoting and stress-buffering effects of early relational health (ERH) and calling for universal ERH promotion in pediatric primary care. However, little educational content for the observation and promotion of ERH is available, highlighting the need for scalable ERH training modules.

**Objective:**

This study aims to investigate the acceptability, feasibility, and impact of the “Lens of Emotional Connection,” a self-paced, asynchronous, ~45-minute ERH training module codeveloped by Reach Out and Read and the Center for Early Relational Health at Columbia University. The module introduces practitioners to emotional connection, an observable component of ERH, through written and video didactic content and experiential rating of emotional connection in videos of parent-child dyads interacting face-to-face.

**Methods:**

The evaluation was conducted by the Carolinas Collaborative. Pediatric providers across 8 clinical sites were invited to participate and responded to embedded pre-post surveys. Focus groups conducted with participants further examined the educational experience.

**Results:**

Of 653 invited clinicians, 207 (31.7%) participated in the module. Individual survey responses were available for 44-75 participants, depending on the question. Of responders, 64 out of 69 (93%) reported the module was a good way to learn about emotional connection, and 63 out of 69 (91%) felt the module provided valuable knowledge. Overall, 60 out of 69 respondents (87%) reported satisfaction with the module length, and 36 out of 44 respondents (82%) reported they would recommend this training to other clinicians. Focus groups echoed these findings. Comparison of pre-post data showed the greatest changes were in familiarity with emotional connection (n=75, pre mean 54.20, SD 18.59; post mean 73.99, SD 14.73; Cohen *d*=1.14; *P*<.001) and confidence in observing the quality of the parent- or caregiver-infant relationship during well-child visits (n=75, pre mean 55.36, SD 18.49; post mean 74.20, SD 14.07; Cohen *d*=1.28; *P*<.001). Suggested areas for improvement included more thorough explanations of specific components of emotional connection identified in parent-child interaction videos, a desire for synchronous live training, and additional content addressing what to do if low emotional connection is identified.

**Conclusions:**

In this evaluation of a training module designed to introduce pediatric practitioners to ERH and emotional connection, acceptability among participants was found to be high, with most responders reporting it as valuable and reporting they would recommend it. Statistically significant impact was noted in both perceptions of the importance of information about emotional connection and perceived knowledge acquisition. Feasibility of widespread implementation with voluntary participation, as here, was relatively low, with only a minority completing the module. Critically, Reach Out and Read’s commitment to iterative creation, validation, and eventual delivery of ERH training creates a scalable avenue for wide-scale implementation, given the organization’s presence in >6500 clinics across the 50 states.

## Introduction

The intricate process of brain development occurs most rapidly in the first 3 years of life and depends on the complex interplay of a child’s biological and emotional responses to both their physical environment and early relationships [1]. Early life exposure to prolonged, frequent, or severe stressors without the buffer of a supportive caregiver puts children at significant risk for poor health outcomes and developmental delays [2], including in social-emotional development, which the American Academy of Pediatrics (AAP) includes as a key domain for surveillance and screening in its Bright Futures Guidelines [3]. Strategies that promote positive parent-child interaction can mitigate these stressors and improve developmental outcomes [4,5]. Given the importance of these positive experiences, the AAP released a policy statement in 2021 on preventing childhood toxic stress that emphasized the importance of promoting early relational health (ERH) in pediatric primary care [5]. Contrasting this recommendation, little educational content for the observation and promotion of ERH is available, and consequently, most clinicians do not receive training in ERH in medical school or residency programs. This creates a clear gap in consistent assessment and anticipatory guidance in the domains of social-emotional development, which is exacerbated for children and families from minoritized populations [6,7].

There is therefore an urgent and critical need for the development and evaluation of scalable educational resources about the importance of ERH, particularly observable elements such as emotional connection, a key indicator of ERH associated with improved child outcomes [8,9]. Observing and engaging in this domain during clinical care can help providers gain insight into a young child’s relationships, offer opportunities for light-touch interventions that strengthen those relationships (eg, encouraging talking, singing, playing, and reading), and help identify families who may need additional resources to support them and their young children [10]. Previous studies have shown that training in this area can improve residents’ ability to identify different levels of emotional connection and improve their attitudes about the importance of emotional connection discussions during primary care visits [11].

Reach Out and Read as an avenue for wide-scale implementation of ERH promotion in pediatric primary care is specifically referred to in the AAP policy statement. Reach Out and Read is an evidence-based literacy intervention that builds on the unique relationship between caregivers and pediatricians to encourage interactions with children through early language and literacy to nurture strong relationships. Families participating in Reach Out and Read are more likely to read aloud to their children, leading to improved language scores compared to controls, an effect consistent across families of diverse backgrounds [12-15]. Reach Out and Read has consequently been identified as a primary component of pediatric primary care and endorsed as essential by the AAP [16], especially owing to the wealth of research demonstrating that shared reading is instrumental in promoting healthy social-emotional development [17-19]. Given the evidence supporting the literacy effects of Reach Out and Read and its impact on increasing the frequency of shared reading, layering in additional components that promote ERH into the Reach Out and Read curriculum is an especially promising avenue to support scalable implementation of ERH promotion in pediatric primary care.

Here, the Carolinas Collaborative, in partnership with Duke University, investigated the acceptability, feasibility, and impact of an early version of a self-paced, asynchronous, ~45-minute ERH training module codeveloped by Reach Out and Read and the Center for Early Relational Health at Columbia University. The module addresses the observation of emotional connection within the pediatric well-child visit as a core component of ERH and child development. The Carolinas Collaborative was established in 2016 as an AAP Community Pediatrics Training Initiative collaborative and is a coordinated collaboration between 8 academic institutions and health care systems serving as systems change infrastructure in North and South Carolina for innovation, training, research, and advocacy best practices to drive scalable child public health solutions. Pediatric clinicians from 8 clinical sites were invited to complete the module via email. Through pre-post surveys and focus groups, the research team investigated the impact of this new ERH module on perceived knowledge and attitudes about emotional connection and gained insight into additional training strategies to inform future design.

## Methods

### Training Module

Training content was based on a validated laboratory measure of the strength of emotional connection in parent-child dyads [8,20] adapted for use in the clinical setting through an iterative process that began with a learning collaborative between Reach Out and Read and the Center for Early Relational Health at Columbia University, and also included pediatric providers (spanning pediatricians, family physicians, and nurse practitioners) and 2 parent leaders. The training module was hosted on Reach Out and Read’s Pathwright portal but was only available by special invitation. Training began with an experiential introduction to the “Lens of Emotional Connection” through four ~30-second videos of mother-infant dyads interacting with one another face-to-face, with varying levels of emotional connection across the videos. Participants were asked to use their “gut feeling” to rate the strength of emotional connection using visual analog scales from ‘not emotionally connected’ to “emotionally connected” (range 1‐100, but no numbers appearing for participants). Videos of these at-home interactions were recorded via Zoom as part of the longitudinal COMBO Initiative [21] and represent ethnically diverse mother-infant dyads at 4‐6 months postpartum. Participants were then provided with written and video educational content of the principles of emotional connection, followed by the same 4 plus 3 new mother-infant videos for practice rating the strength of emotional connection using the same visual analog scales. Participants were informed that the scale acts as an educational tool and that they are not expected to use such a scale in their clinical practice. Finally, participants were shown 3 videos of parent-infant well-child visits with Reach Out and Read pediatricians trained in the observation and promotion of emotional connection for a real-life demonstration of using the new “lens” in a clinical setting. The training module was estimated to take 45 minutes to complete.

### Mixed Methods Study Design

To evaluate the training module, this project used a sequential mixed methods design [22] with a pre-post survey process and follow-up focus groups. Surveys permitted a breadth of understanding across a larger number of participants; focus groups provided explanatory information to further understand and explain survey data as well as to identify relevant factors that were not captured in surveys. This mixed methods study is reported in accordance with both GRAMMS (Good Reporting of a Mixed Methods Study) and STROBE (Strengthening the Reporting of Observational Studies in Epidemiology) reporting guidelines to ensure proper reporting of both quantitative and qualitative aspects. The datasets generated and analyzed during this study (focus group interviews and survey data) are not publicly available, as the approved institutional review board (IRB) protocol did not allow for this. However, data can be made available by the corresponding author on reasonable request and pending appropriate IRB approval.

### Survey

Pre-post surveys were developed across a series of recurring meetings held with an interdisciplinary team that included module developers, academic pediatricians, and social scientists with evaluation and measurement expertise. The pre- and postsurvey rationale was chosen to measure the effectiveness of the training in improving participants’ perceived confidence and knowledge in ERH. Additional postsurvey items assessed module experience. Survey development and review by the diverse members of the planning team, which included experts in ERH, pediatricians with experiential engagement with pediatrics training, and training intervention developers, focused on content and face validity to ensure relevance, clarity, and alignment with module objectives. Surveys are available in Multimedia Appendix 1.

The presurvey questions were administered before participants engaged with module training content and assessed age, self-identified race and ethnicity, and gender, training level, and questions about previous ERH and emotional connection experience, attitude, and knowledge. The postsurvey questions were administered after participants completed all module training content and included parallel items addressing attitude and knowledge related to ERH and emotional connection (repeated from the presurvey), as well as items addressing module experience and perspective on the implementation of emotional connection-related practices in pediatric primary care. Pre- and postsurveys were programmed and hosted on Columbia University’s Qualtrics and integrated directly into the Pathwright module experience. The combined length of the training module and pre-post surveys was ~45 minutes.

### Enrollment

A total of 653 residents, advanced practice providers, and faculty at all 8 sites of the Carolinas Collaborative were recruited to participate in this study between June and October 2023 (Table 1). Inclusion criteria required participants to be either attending clinicians, resident clinicians, or faculty. Recruitment was the same across all sites, via email invitation with a link to the training. The module could be done on any computer and whenever they found or were allotted time. (Some clinical sites provided protected time for residents to complete the modules and surveys.) Participants were able to begin and finish at a later time as long as they used the same computer. It was made clear to participants that all aspects of this study were voluntary, and that participants could complete the training and choose to have their responses used in research or opt out of the use of their data for research purposes.

**Table 1.** Eligible participants invited to the study. Individuals invited to participate in the modules and study were largely residents and represented all 8 Carolinas Collaborative pediatrics programs.

Site	Faculty, n	Residents, n	Total, n
Prisma Health-Midlands	10	51	61
Prisma Health-Upstate	20	56	76
MUSC^a^	16	73	89
UNC^b^	19	70	89
Atrium Wake Forest Baptist Health	12	62	74
Atrium Health	15	38	53
ECU^c^	18	56	74
Duke	39	98	137
Total	149	504	653

a MUSC: Medical University of South Carolina.

b UNC: University of North Carolina.

c East Carolina University.

### Data and Statistical Analysis

Survey data were not available for all participants who engaged with the module due to a combination of skipped survey items, some participants stopping before the postsurvey, and technical errors in the integration of data across Qualtrics and Pathwright. Sample sizes for available data for each survey item ranged between 44 and 75. Available data were analyzed using SAS software (version 9.4; SAS Institute, Inc.; copyright 2002‐2012) and used descriptive statistics and paired 2-tailed *t* tests to address change for individuals with both pre- and postdata. Additional ordered logit and ordinary least squares regression models examined the potential effect of varied participant demographic characteristics and prior related experience; however, minimal effect of background characteristics was detected, and these results are thus not reported here.

### Focus Groups

Individual Carolinas Collaborative sites coordinated with the social science research team at Duke University to schedule focus group opportunities with participants who completed the training between August and November 2023. Using a sequential mixed methods design [22], focus groups were constructed to provide additional context and explanation for survey-based quantitative results, in addition to providing added nuance more generally on experience with and effect of module engagement. Participants were recruited via email sent to the same distribution lists as during initial recruitment, and an incentive of a US $10 gift card was provided for participation in focus groups. As a result, 7 semistructured focus groups were conducted with 33 total participants across 4 Carolinas Collaborative sites. Focus groups were held virtually, lasted approximately 1 hour, and assessed initial knowledge and perspective on emotional connection, experience with the module, use, value of the module for their knowledge and practice, and opportunities for improvement; see Multimedia Appendix 2 for the focus group guide. Recruitment concluded after the interested participants identified by the end of the planned data collection period had been engaged. While the team had not definitively reached saturation across all possible points, results were revealing clear common themes regarding priority areas of experience and outcomes. Decisions against added waves of recruitment were based on project timeline considerations, likelihood of more limited participation with added recruitment efforts, and value in the existing scope data that had been collected (eg, clear common themes identifiable based on data already collected). Focus groups were recorded and transcribed. Data were analyzed using a rapid analysis framework [23], identifying key themes in responses to questions addressed. This used templated matrices structured based on primary areas of the focus group instrument (eg, aspects of module experience, distinct outcomes; see Multimedia Appendix 3 for matrix structure), review of transcripts and documentation of relevant content per item in each focus group, and cross-review among analysts (members of the social science team) to identify themes per topic. Qualitative and quantitative results were considered in tandem, with qualitative results providing an added explanatory lens on quantitative findings.

### Ethical Considerations

The survey portion of this study was approved by the AAP’s IRB (#22 ER 01), which served as the main approval body; the approval notice was shared with and reviewed by each constituent IRB within the Carolinas Collaborative (Atrium Health Levine Children’s Hospital, Atrium Health Wake Forest Baptist, Duke University, East Carolina University, Medical University of South Carolina, Prisma Health Midlands, Prisma Health Upstate, and the University of North Carolina), some of which did not require further review and some which considered the study exempt following site-specific application. The Columbia University IRB also separately approved the study (#AAAU5749). Participant consent was waived by both the AAP and Columbia University IRBs because the data collected were anonymous and could not be linked to any identifying information. In lieu of consent, all participants were provided with an informational sheet (electronically before the survey) that provided them with all the information about the study and offered them the opportunity to opt in or out of research participation. Participants could complete the training without participating in the research study. Participants did not receive compensation for their time completing the training (though, as noted above, a US $10 gift card was offered to the subset of participants who took part in the focus groups). The videos used in the training itself were collected in a previous study with participants who gave consent for their videos to be used for research and training purposes. The focus group portion of this study was approved by the Duke University IRB (#2023‐0300).

## Results

### Participants

Of those invited, 207 (31.7%) clinicians participated in the module, and of these participants, 117 respondents completed at least 1 survey (56.5% response rate) with representation across 8 Carolinas Collaborative sites (Table 2). Participants were mostly under 30 years old (n=71, 60.7%), self-identified as women (n=92, 78.6%), and self-identified as White or Caucasian race and ethnicity (n=92, 78.6%). Most participants were residents (n=38, 32.5% post graduate year (PGY)-1; n=33, 28.2% PGY-2; n=23, 19.7% PGY-3/4). While response rates could not be quantified among faculty versus residents due to the inherent lack of demographic data for participants who did not complete at least 1 survey, participation rates relative to invitations were relatively similar for the 2 groups among the 115 participants who reported on their clinical role: n=19 of 149 (12.8%) for faculty and n=94 of 504 (18.7%) for residents. While the majority (n=108, 93.2%) reported having completed the Reach Out and Read Core Module, only 26.4% (n=31) reported having moderate or significant prior experience with emotional connection.

**Table 2.** Study participant characteristics (n=117).

Characteristics^a^	Participants, n (%)
Age (years)	
Under 30 years old	71 (60.68)
30 years old or older	45 (38.46)
Missing response	1 (0.85)
Gender	
Man	22 (18.80)
Woman	92 (78.63)
Nonbinary	1 (0.85)
Prefer not to answer	2 (1.71)
Missing response	0 (0)
Race and ethnicity	
Asian or Pacific Islander	5 (4.27)
Black or African American	6 (5.13)
Latino or Latina	4 (3.42)
Multiracial	9 (7.69)
White or Caucasian	92 (78.63)
Missing response	1 (0.85)
Role	
AAP^b^ (eg, NP^c^ and PA^d^)	2 (1.71)
PGY^e^-1	38 (32.48)
PGY-2	33 (28.21)
PGY-3/4	23 (19.66)
Physician - private panel	1 (0.85)
Physician - resident preceptor and private panel	11 (9.40)
Physician - resident preceptor only	7 (5.98)
Other	0 (0)
Missing response	2 (1.71)
Carolinas Collaborative locations	
Atrium Health - Levine	5 (4.27)
Atrium Health - Wake Forest University	23 (19.66)
Duke University	18 (15.38)
East Carolina University	12 (10.26)
Medical University of South Carolina	16 (13.68)
Prisma Health-Midlands	27 (23.08)
Prisma Health-Upstate	8 (6.84)
UNC^f^ Chapel Hill	6 (5.13)
Missing	2 (1.71)
Amount of prior experience with emotional connection	
I have no experience with this	21 (17.95)
I have a little experience with this	65 (55.56)
I have a moderate amount of experience with this	26 (22.22)
I have a lot of experience with this	5 (4.27)
Missing response	0 (0)
Prior experience type (n=136^g^)	
Involved in ROR^h^ training development	2 (1.71)
Prior experience via Reach Out and Read Core module	108 (92.31)
Prior experience via Keystones of Development curriculum	19 (16.24)
Missing response	7 (5.98)

a All demographic and personal characteristics were self-identified by respondents.

b AAP: American Academy of Pediatrics.

c NP: nurse practitioner.

d PA: physician assistant.

e PGY: postgraduate year.

f UNC: University of North Carolina.

g Respondents were instructed to select “all that apply” for this question, resulting in 136 responses to this question.

h ROR: Reach Out and Read.

### Module Experience

Survey data indicated that 64 out of 69 respondents (93%) reported that the module was a good way to learn about emotional connection (responses ranging between “somewhat agree” and “strongly agree”; Table 3). More than half of these respondents (n=40, 58%) reported they either “agree” or “strongly agree.” Similarly, 63 out of 69 respondents (91%) reported that the training provided valuable knowledge (responses ranging from “somewhat agree” to “strongly agree”; Table 3), with 39 (57%) reporting agreement to some degree and 24 (35%) reporting strong agreement. In addition, 60 out of 69 respondents (87%) reported satisfaction with the module length. Focus group data echoed a positive experience with the module content and format, with focus group data indicating that the videos embedded within the modules provided valuable examples of what to look for when assessing emotional connection:

The content was easy to understand and very well laid out. Yeah, I think I particularly liked how interactive the videos were and the prompts after.

…the online format was good because you got to see videos of actual children. [If] we just did like a role-playing thing or someone presented it then you might miss out on that aspect.

A plurality of respondents reported that the training provided valuable knowledge, was a good way to learn about emotional connection, and presented strategies that could be implemented.

Suggested areas for improvement, reflected in both an open-ended survey question and in focus group data, frequently addressed aspects of content structure within the module. This included a desire for the incorporation of written summaries and/or reviews of the video content by experts either within or following module completion and clearer explanations of specific behaviors and examples of emotional connection within each video. Focus group data also indicated technical challenges with the log-in process, some challenges with the repetitive nature of the module videos, response fatigue, and a desire for added diversity in family structure within the videos (ie, the inclusion of more dyads beyond mother-baby):

I do remember it was initially kind of hard to figure out how to log in.

When the same video came up I’m like okay, I saw this.

I feel like maybe it would have been better to have the different types of caregivers present with them there, like instead of just mom, since that’s like more what we see in real life, [the] different spectrum of caregivers.

Focus group data additionally explored the experience of how the module was presented to participants and when respondents were expected to complete the modules. Some respondents indicated that their programs set aside a specific time for module completion, while others reported completing it independently, with benefits for each format:

Doing it with the dedicated time..., I was more focused because that’s what everybody was doing.

You can do it at like your own pace, like kind of a game, review the videos maybe a second time if you want to. Everyone has their own phase of like completing the things.

**Table 3.** Value and acceptability of the training module.

Value and acceptability items	Respondents, n (%)
The training gave valuable knowledge (n=69)	
1 (strongly disagree)	1 (1.45)
2	0 (0)
3	1 (1.45)
4 (neither agree nor disagree)	4 (5.80)
5	21 (30.43)
6	18 (26.09)
7 (strongly agree)	24 (34.78)
The module was a good way to learn about emotional connection (n=69)	
1 (strongly disagree)	1 (1.45)
2	1 (1.45)
3	1 (1.45)
4 (neither agree nor disagree)	2 (2.90)
5	24 (34.78)
6	14 (20.29)
7 (strongly agree)	26 (37.68)
The strategies presented through this training could be implemented in respondents’ practice (n=69)	
1 (strongly disagree)	1 (1.45)
2	0 (0)
3	0 (0)
4 (neither agree nor disagree)	2 (2.90)
5	20 (28.99)
6	21 (30.43)
7 (strongly agree)	25 (36.23)
Length of the training (n=69)	
Too short	0 (0)
Just about right	60 (86.96)
Too long	9 (13.04)
Would recommend training (n=44^a^)	
No	2 (4.55)
Unsure	6 (13.64)
Yes	36 (81.82)
Recommendations on Incorporating into residency training (n=44^a^)	
Don’t know	0 (0)
Would not recommend in residency training	0 (0)
Recommend making available but not promoting	2 (4.55)
Recommend (not require)	24 (54.55)
Require for all residents	17 (38.64)
Other	1 (2.27)

a The smaller sample size represents attrition between survey initiation and survey completion.

However, challenges were identified in both approaches: set-aside time may be rushed or pulled from busy schedules, but finding one’s own time could also be challenging. Distractions were experienced during module completion in both cases.

Regarding the module’s future use, 36 out of 44 respondents (82%) reported they would recommend this training to other clinicians. In addition, most respondents suggested this module be required (n=17 out of 44, 39%) or recommended (n=24 out of 44, 55%) as part of residency training. An additional 2 out of 44 respondents (5%) indicated it should be available but not promoted, and 1 respondent who selected an “other” response indicated this should not exist as a stand-alone activity but rather should be “incorporated into a larger/longer learning session where the nuances of such strategies can be explored through discussion”; this was echoed in focus group data, which indicated a desire for synchronous or live training or an opportunity for more interpersonal engagement through the training:

It would have been cool if there was like an adaptive strategy to make it more of like a group-based activity so those of us who were in the same room could have done it more, in an engaged manner.

### Participant Effect and Implications for Practice

Additional survey data comparing responses on assessed outcomes before and after module completion (Table 4) indicated that the greatest changes were in familiarity with the concept of emotional connection (n=75, pre mean 54.20, SD 18.59; post mean 73.99, SD 14.73; Cohen *d*=1.14; *P*<.001) and confidence in observing the quality of the parent- or caregiver-infant relationship during well-child visits (n=75, pre mean 55.36, SD 18.49; post mean 74.20, SD 14.07; Cohen *d*=1.28; *P*<.001). In qualitative data from focus groups, respondents reported value in varied respects, including having a framework and model that provides guidance for the observation of emotional connection and that demonstrates the varied ways emotional connection can present and be observed:

It was useful because it gave like a practical framework on how to work with patients and also make a relationship with them in the office, especially with you maybe just first meeting these people and their baby in a more technical way.

**Table 4.** Pre-post measures of module intended outcomes.

Outcome measures	Presurvey, mean (SD)	Postsurvey, mean (SD)	*t* test (*df*)		*P* value^a^	Effect size (Cohen *d*)^b^	Respondents, n^c^
Estimated minimum time to observe the quality of the relationship between a parent/caregiver and infant during a well-child visit (range 1‐120 min)	13.14 (18.42)	8.11 (14.98)	1.98 (70)		.05	0.23	71
Familiarity, confidence, and beliefs about early relational health in well-child visits (scale=1‐100)							
Familiarity with the concept of emotional connection	54.20 (18.59)	73.99 (14.73)	−9.9 (74)		<.001	1.14	75
Confidence in observing the quality of the parent- or caregiver-infant relationship during well-child visits	55.36 (18.49)	74.20 (14.07)	−11.09 (74)		<.001	1.28	75
Belief that understanding early relational health is essential to work as a clinician	80.41 (15.72)	86.00 (14.20)	−3.92 (64)		<.001	0.49	65
Belief that observing emotional connection in well-child visits will improve practice as a clinician	80.91 (15.73)	84.15 (14.47)	−2.23 (45)		.03	0.33	46
Belief that observing emotional connection in well-child visits will improve outcomes for patients and their families	78.34 (16.01)	82.68 (16.02)	−2.39 (46)		.02	0.35	47

a All *P* values are based on 2-tailed tests.

b Effect size (Cohen *d*): no effect=0.0‐0.19, small=0.2‐0.49, medium=0.5‐0.79, large=0.8 and above; calculated as *d=t/√n*

c Analyses are subset to only include respondents with both pre and post responses.

I really liked having, you know, all the different examples of parent interaction…the model of what is good doesn’t necessarily look exactly the same for everyone.

Significant pre-to-post change was evidenced across all survey measures related to familiarity, confidence, and beliefs about ERH and emotional connection in well-child visits, and the expected estimated minimum time to observe the quality of the relationship between a parent-caregiver and infant during a well-child visit decreased.

Beliefs about ERH also exhibited a statistically significant change between the pre- and postsurvey. Specifically, participants exhibited a change in their beliefs that understanding ERH is essential to their work as a clinician (n=65, pre mean 80.41, SD 15.72; post mean 86, SD 14.20; Cohen *d*=0.49; *P*<.001), observing emotional connection in well-child visits will improve their practice as a clinician (n=46, pre mean 80.91, SD 15.73; post mean 84.15, SD 14.47; Cohen *d*=0.33; *P*=.03), and observing emotional connection in well-child visits will improve outcomes for patients and their families (n=47, pre mean 78.34, SD 16.01; post mean 82.68, SD 16.02; Cohen *d*=0.35; *P*=.02). As one focus group respondent elaborated:

It makes me pay attention more even like with these little, very little babies that the emotional connection between them and their caregivers really does have such a huge impact.

Of note, the baseline (pre) data indicated considerably greater agreement (mean ranges 78.34‐80.91) with these assessed beliefs about emotional connection compared to assessed confidence in or familiarity with emotional connection (mean ranges 54.20‐55.36) prior to module engagement. This suggests the training is filling a learning gap that the respondents desire to gain expertise in.

### Barriers to Integrating Emotional Connection Into Practice

In survey data, 66 out of 69 respondents (95%) reported that the strategies presented could be implemented in clinical practice (“somewhat agree” to “strongly agree” that they could be implemented) (Table 3). When asked to consider primary challenges to implementing the tools taught in the module in practice, the time required to assess emotional connection during a clinical encounter was the most noted challenge (30 out of 188 survey responses [25.6%] on a “select up to 3 response” item) (Table 5). As one focus group participant explained:

Time is always a barrier. Time is always against you in clinic, you know, trying to get to the next patient, the next patient, the next patient…I’d love to spend as much time as possible like observing and talking about it, but a lot of times it’s like I’ve got to tell them about their vaccines. I’ve got to talk to them about like lab testing, you know.

**Table 5.** Primary barriers to integrating emotional connection into practice (N=188^a^).

Barriers	Respondents, n (%)
Clinic support or capacity for supporting families	9 (7.69)
Cultural differences between me and my patients and families	14 (11.97)
Families may not be open to talking about this	13 (11.11)
Knowing what to do if I identify a problem	19 (16.24)
Knowledge of how to do this	5 (4.27)
Language differences between me and my patients and families	15 (12.82)
The focus and energy required to do this	8 (6.84)
Time required to do this	30 (25.64)
Other	2 (1.71)
Did not respond to question	73 (62.39)

a Respondents were instructed to select up to 3 responses for this question; therefore, the total N of responses for this question is greater than the number of survey respondents.

Respondents most often reported implementation challenges associated with the time required to assess emotional connection, knowing what to do if a problem was identified, and language and cultural challenges.

In addition, 19 out of 188 survey responses (16.2%) pointed to potential challenges with determining follow-up action steps (‘Knowing what to do if I identify a problem’), with focus group data echoing this:

I know how to realize good and bad. I know what to do with the good ones, but like what exactly should I do if I’m like ‘you’re not connecting to your child’?

Language and cultural differences between respondents and their patients and families were also cited as potential challenges (15 out of 188 survey responses [12.8%] and 14 out of 188 survey responses [12%], respectively), in addition to family willingness to engage in relevant discussion (13 out of 188 survey responses [11.1%]) (Table 5). Focus group data additionally raised the concern that well-child visits may not provide a good example of family dynamics at home:

Taking off the clothes, and it’s cold, and there are strangers…it feels a little bit harsh to judge what someone’s like home interaction with their baby is like based on me walking in for five minutes.

We see a lot of families that speak other languages and a lot of the times maybe the parents focusing on like the interpreter on the computer so we’re not always getting a full kind of natural reaction between the parent and the kid…also if we have people coming in with maybe a grandparent or some other guardian who’s not their parent or not even the person who they live with primarily, we’re not always getting a feel for what the connection is with the people they live with.

## Discussion

### Principal Findings

ERH and emotional connection have been identified as foundational components of healthy child development, and yet there is a lack of scalable training resources to incorporate the observation of ERH into pediatric primary care practice [4,24-28]. Efforts to date have primarily focused on diagnosing and treating relational problems in children with relatively severe disturbances in behavior [29]. In addition to being narrowly targeted and costly, these efforts do not lend themselves to universal primary prevention [30]. Indeed, in a recent study exploring residency program directors’ views on the adequacy of current ERH training, a clear gap in this area of medical education was identified [31].

Here, we tested the feasibility of a brief self-paced, asynchronous, interactive ERH training module in teaching pediatricians the observation of emotional connection, an observable component of ERH. We found that the large majority of respondents reported that the information provided in this module gave them valuable knowledge on emotional connection, and that this module was a good way to learn about emotional connection. Despite social-emotional development and healthy relationships being domains addressed in the AAP Bright Futures guidelines and policy statements [5], these results clearly show that this is an area of educational growth needed for clinicians who were initially unfamiliar with these concepts. In addition, specific results provide key areas to target. For instance, pretraining scores for knowledge and confidence (familiarity with emotional connection and confidence in observing parent and caregiver–infant relationships during well-child visits) were lower than scores for beliefs about emotional connection (eg, its importance to clinical work, practice improvement, and patient outcomes). This may explain the larger effect sizes for knowledge and confidence, and it suggests the training addresses a key gap: residents value ERH and emotional connection but have less knowledge and confidence in observing them. This is also consistent with prior literature indicating knowledge and confidence to be more responsive to short trainings than beliefs and attitudes [32,33].

In a field as complex as medicine, evolving medical information necessitates a feasible and acceptable method for efficiently disseminating new knowledge. In recent years, asynchronous modules have been thought of as an efficient way to reach learners in different locations [34,35]; additionally, with time constraints on faculty and residents, modules developed by topic experts can be a valuable way to add to clinician education without coordinating timing for in-person learning. Access to modules can decrease the burden on individual programs or faculty to find ways to accomplish educational and curricular goals in both training and continuing medical education. Modules and other e-learning strategies have long been considered effective in accomplishing this goal [36,37] and were widely adopted as a key component of medical and graduate medical education during the COVID-19 pandemic [38,39]. In the domain of ERH, there is a clear benefit to these modules, which enable multiple learners across multiple programs to be trained in a similar manner, allowing for shared knowledge and a common language to discuss social-emotional health and development.

However, there is significant variability in the content and quality of medical education training modules. Intentional development of modules through interdisciplinary collaborations like the one presented here can potentially increase access to high-quality educational opportunities, especially for new or emerging topics. The results of this pilot training suggest that a~45-minute training module is a feasible and acceptable way to provide education about ERH and specifically emotional connection, and that providers found the training valuable to improve their knowledge base.

In addition to the knowledge that these modules provide and an acceptable means of delivering novel educational content, it is also important to look at the challenges participants face. Notably, we discovered challenges with identifying time to complete modules, and the benefits and drawbacks of asynchronous delivery are critical to consider. Additionally, concerns noted by participants about how to incorporate learned skills into already full well-child visits are pertinent to many topics as more components are introduced into the well-child visit. Given these challenges, it will be essential to develop trainings that address how to easily integrate the content into the workflow and how to make changes or adjustments to clinical practice. These benefits and challenges are generalizable to a wide range of topics and disciplines that are interested in expanding educational opportunities via asynchronous modules.

### Limitations and Future Directions

This study has several limitations, many of which can direct us to future opportunities for investigation. The first is that there may have been recruitment bias. Participants who engaged with the voluntary training module and study may have been interested in emotional connection or closely related topics that inspired them to participate. Response bias may also be a factor; specifically, social desirability in reporting knowledge or positive attitudes toward the subject or for residents who were asked to complete a module by their preceptors, regardless of the voluntary nature of the study [40]. Qualitative data provided some nuance on this possible bias. Although focus group participants provided an overall positive response to the module, they also displayed freedom to critique the training in this new feedback format, indicating that, indeed, the forced-choice survey data might be slightly inflated. Additionally, while there is value in the fact that these data represent 8 unique sites across the Carolinas Collaborative, they are all geographically located in the same area of the country, which limits generalizability. There was also a lack of diversity within the demographics of the participating clinicians. Additionally, adult learning styles were not considered for participants, and nuances in learning styles may make this training method more or less effective for individuals. Finally, this learning modality was not compared to a more traditional in-person experience, which was suggested by some participants as important and desired. We also do not have information from participants who started but failed to complete the modules. While we obtained some information from focus groups to understand improvements that could be made to the module, it would be important to better understand why some participants did not complete the training. Future studies will also need to evaluate if participation rates and satisfaction scores differ by sites that provide protected time for completion of the module versus those that do not.

Second, the study had a relatively low response rate. The timing of the study might have been a limiting factor in our successful recruitment of participants. Due to the timing of funding availability, modules were released at the beginning of June, and recruitment continued through October. Because the study primarily targeted residents and teaching faculty, timing may have contributed to the low response rate, as this is generally not an ideal time for change or new initiatives in academic centers. The voluntary nature of the training also likely contributed to the low response rate. This points to the necessity of embedding future iterations of such modules into already existing training infrastructure, such as the Reach Out and Read Core Curriculum, making it a mandatory component for certification.

In addition to these limitations, there are several areas that were not addressed by this study that call for future investigation. A concern mentioned by participants in both surveys and focus groups was that while there was thorough education about the importance of and strategies for identifying emotional connection, the modules did not address what to do when a lack of emotional connection was noted or an additional problem with social-emotional development was identified. Along this same theme, more investigation is needed to effectively address cultural and linguistic differences in parenting that could be perceived as “problems” without an appropriate appreciation for different cultural lenses to assess connection. Another future domain of study is how this education impacts clinicians’ daily practice. This could be accomplished by direct observed exams, which are the gold-standard in resident education to assess trainees’ competencies in various aspects of care [41,42]. Finally, we did not address the efficacy of the module, as defined in the ability to change how observers rate the video interactions compared to trained researchers.

Using the information learned here, we have created an updated iteration of this training module that is currently being piloted at Columbia University. This new version expands the training with a higher number of parent-child videos across a larger age range (0‐3 years), incorporates real-time feedback of participants’ ratings of emotional connection, and gives additional opportunities to score videos when the original score was out of range. The new module also includes several suggested actionable next steps if emotional connection is observed to be low, though participants are cautioned that light-touch intervention development targeting emotional connection is a current area under investigation.

### Conclusions

ERH and emotional connection are foundational to healthy child development, and education about these topics delivered through asynchronous digital modules is highly valued by participants. While completion rates of voluntary training are generally low, including in this study, Reach Out and Read’s commitment to creation, validation, and eventual delivery of ERH training creates a scalable avenue for wide-scale implementation, given the organization’s presence in >6500 clinics across the 50 states. The data presented here inform next steps in the iterative design of medical education on ERH. Further research is needed to more fully understand how this training impacts clinician behaviors during clinical encounters, how families receiving care perceive these changes, the impact on child and family outcomes, and potential disparities in the perception of different cultural norms in parenting as demonstrative of strong or weak emotional connection, to avoid over- or underidentification of deficits in this area.

## Supplementary material

10.2196/81250 Multimedia Appendix 1 Pre- and postsurveys.

10.2196/81250 Multimedia Appendix 2 Focus group guide.

10.2196/81250 Multimedia Appendix 3 Matrix structure for qualitative rapid analysis.

## Abbreviations:

AAP: American Academy of Pediatrics
ERH: early relational health
GRAMMS: Good Reporting of a Mixed Methods Study
IRB: institutional review board
STROBE: Strengthening the Reporting of Observational Studies in Epidemiology
