# Supplementary material for: Implementation of Emotional Connection Training in Pediatric Primary Care: Mixed Methods Study
Source: JMIR Med Educ. 2026 Jun 16;12:e81250. doi: 10.2196/81250 (PMC13271710; doi:10.2196/81250)
Supplement: Multimedia Appendix 1 [file mededu-v12-e81250-s001.docx]

**Pre and Post Surveys**

**Pre-Survey**

**Start of Block: INFO SHEET**

**The Lens of Emotional Connection Project**

Thank you for your interest in joining our Emotional Connection project. This is an innovative project piloting a training on the observation and promotion of emotional connection between dyads of parents/caregivers and young children with the goal of helping pediatric care providers partner with the families they serve to support healthy relationships.

We are evaluating our 40-minute CME- and MOC-2-accredited training that is designed to introduce you to a “lens” that can help you in observing the emotional connection between the parents/caregivers and the young children you serve. This training includes anonymous pre- and post-surveys, each of which should take approximately 5 minutes.

**All survey responses are anonymous and will be used to improve the training.** **You can choose whether you would like your survey responses to also be used in our research study.**

If you decide to take part in the research study, no additional surveys are necessary. Your responses will be collected by Qualtrics and stored by Columbia University in New York City. In order to conduct analyses and evaluate the data, survey data will be shared with different universities and programs (including Duke University) who are collaborating with Columbia University. If you have any questions about this research study, please reach out to Principal Investigator Dr. Dani Dumitriu, MD, PhD, at dani.dumitriu@columbia.edu (Study Name: Early Relational Health Training and Evaluation, Protocol #  AAAU5749)

Being in a research study is completely voluntary. You can choose not to be in this research study. Deciding not to be in the research study will not affect your standing within your residency program or professional standing and any supervisors you may have will not know if you participated or not.

Please select one of the following:

- My anonymous survey responses can be used for research purposes
- My anonymous survey responses cannot be used for research purposes

**End of Block: INFO SHEET**

**Start of Block: T1**

The questions below will be used to create a unique ID for yourself; this will be used to link your responses to future surveys.

What month was your oldest parent born in (01=January to 12=December; if this does not apply to you please select "14")

- 01
- 02
- 03
- 04
- 05
- 06
- 07
- 08
- 09
- 10
- 11
- 12
- 14

| 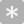 |
| --- |

What are the last 2 digits of your cell phone number?
Please enter a number from 00-99

________________________________________________________________

| 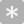 |
| --- |

What are the two digits of the day you were born
Please enter a number from 01-31

________________________________________________________________

| 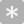 |
| --- |

How many younger siblings do you have?
Please enter a number from 00 to 15

________________________________________________________________

| 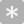 |
| --- |

Write the LAST letter of your youngest parent’s FIRST name.
Please enter a letter from A to Z; If this does not apply to you please type "A"

________________________________________________________________

**End of Block: T1**

**Start of Block: ERH**

Please briefly note any specific trainings or resources you have engaged with on the topic of early relational health.
Please select all that apply

- ROR core module
- Keystones of Development Curriculum
- I have been involved in the development of this training
- Other (specify) __________________________________________________

How would you rate your prior experience/training around early relational health?

- I have no experience with this
- I have a little experience with this
- I have a moderate amount of experience with this
- I have a lot of experience with this

**End of Block: ERH**

**Start of Block: Pre/Post Questions ROR**

| Page Break |  |
| --- | --- |

The questions below ask about your views as related to emotional connection as a construct within early relational health.

There are no right and wrong answers; we want your honest response on your perspective.

What do you think is the minimum amount of time required to observe the quality of the relationship between a parent/caregiver and an infant (in minutes)?

- I don't know
- 1
- 2
- 3
- 4
- 5
- 6
- 7
- 8
- 9
- 10
- 11
- 12
- 13
- 14
- 15
- 16
- 17
- 18
- 19
- 20
- 21
- 22
- 23
- 24
- 25
- 26
- 27
- 28
- 29
- 30
- 31
- 32
- 33
- 34
- 35
- 36
- 37
- 38
- 39
- 40
- 41
- 42
- 43
- 44
- 45
- 46
- 47
- 48
- 49
- 50
- 51
- 52
- 53
- 54
- 55
- 56
- 57
- 58
- 59
- 60
- 61
- 62
- 63
- 64
- 65
- 66
- 67
- 68
- 69
- 70
- 71
- 72
- 73
- 74
- 75
- 76
- 77
- 78
- 79
- 80
- 81
- 82
- 83
- 84
- 85
- 86
- 87
- 88
- 89
- 90
- 91
- 92
- 93
- 94
- 95
- 96
- 97
- 98
- 99
- 100
- 101
- 102
- 103
- 104
- 105
- 106
- 107
- 108
- 109
- 110
- 111
- 112
- 113
- 114
- 115
- 116
- 117
- 118
- 119
- 120

How familiar are you with the concept of emotional connection?

|  | Not familiar | Very familiar |
| --- | --- | --- |

| 2 | 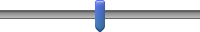 |
| --- | --- |

How confident are you in observing the quality of the parent/caregiver-infant relationship during well child visits?

|  | Not at all | Extremely |
| --- | --- | --- |

| 1 | 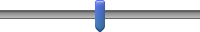 |
| --- | --- |

Please list up to three observable elements that indicate the quality of the parent/caregiver-infant relationship.

- #1: __________________________________________________
- #2: __________________________________________________
- #3: __________________________________________________

| Page Break |  |
| --- | --- |

Please rate your agreement with the following statements

Understanding Early Relational Health is essential to my work as a clinician

|  | Strongly Disagree | Neither agree nor disagree | Strongly agree | I do not know what this is |
| --- | --- | --- | --- | --- |

| 2 | 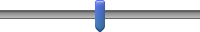 |
| --- | --- |

Observing emotional connection in well-child visits will improve my practice as a clinician.

|  | Strongly Disagree | Neither agree nor disagree | Strongly agree | I do not know what this is |
| --- | --- | --- | --- | --- |

| 3 | 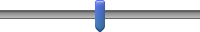 |
| --- | --- |

Observing emotional connection in well-child visits will improve outcomes for patients and their families.

|  | Strongly Disagree | Neither agree nor disagree | Strongly agree | I do not know what this is |
| --- | --- | --- | --- | --- |

| 4 | 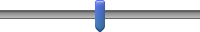 |
| --- | --- |

**End of Block: Pre/Post Questions ROR**

**Start of Block: Background**

These questions give us some background information about you.

Which of the following best describes your racial/ethnic identity?
Select all that apply

- White/Caucasian
- Black/African American
- Latino/a/x
- Asian/Pacific Islander
- American Indian/Alaska Native
- Multiracial
- Prefer to Specify: __________________________________________________
- Prefer not to answer

What terms best express how you describe your current gender identity?
Select all that apply

- Woman
- Man
- Non-binary
- Transgender
- None of these describe me, and I'd like to consider additional options
- Prefer not to answer

*Display This Question:*

*If What terms best express how you describe your current gender identity? Select all that apply = Non-binary*

*Or What terms best express how you describe your current gender identity? Select all that apply = Transgender*

*Or What terms best express how you describe your current gender identity? Select all that apply = None of these describe me, and I'd like to consider additional options*

Are any of these a closer description to your gender identity?

- Transman/Transgender Man/FTM
- Transwoman/Transgender Woman/MTF
- Genderqueer
- Genderfluid
- Gender variant
- Two-Spirit
- Questioning or unsure of gender identity
- None of these describe me, and I want to specify __________________________________________________

How old are you?

- Under 30
- 30-39
- 40-49
- 50 or above
- Prefer not to answer

| Page Break |  |
| --- | --- |

Which region are you from?

- Carolinas Collaborative
- New Jersey
- Florida
- Other (specify) __________________________________________________

*Display This Question:*

*If Which region are you from? = Carolinas Collaborative*

Which Carolinas Collaborative Training Program are you affiliated with?

- East Carolina University
- Duke University
- UNC Chapel Hill
- Atrium Health - Wake Forest University
- Atrium Health - Levine
- Medical University of South Carolina
- Prisma Health – Midlands
- Prisma Health – Upstate
- Other (specify) __________________________________________________

*Display This Question:*

*If Which region are you from? != Carolinas Collaborative*

What type of clinic?
Select all that apply

- Academic
- Private
- Pediatric
- Family Medicine
- Hospital Affiliated
- Urban
- Suburban
- Rural
- FQHC
- Other (specify) __________________________________________________

What is your role?

- PGY-1
- PGY-2
- PGY-3/4
- Physician - Private Panel
- Physician - Resident Preceptor ONLY
- Physician - Resident Preceptor AND Private Panel
- APP (e.g., NP, PA)
- Other (specify) __________________________________________________

**End of Block: Background**

**Post-Survey**

**Start of Block: ID**

The questions below will be used to create a unique ID for yourself; this will be used to link your responses to previous and future surveys.

What month was your oldest parent born in (01=January to 12=December; if this does not apply to you please select "14")

- 01
- 02
- 03
- 04
- 05
- 06
- 07
- 08
- 09
- 10
- 11
- 12
- 14

| 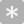 |
| --- |

What are the last 2 digits of your cell phone number?
Please enter a number from 00-99

________________________________________________________________

| 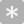 |
| --- |

What are the two digits of the day you were born
Please enter a number from 01-31

________________________________________________________________

| 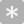 |
| --- |

How many younger siblings do you have?
Please enter a number from 00 to 15

________________________________________________________________

| 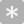 |
| --- |

Write the LAST letter of your youngest parent’s FIRST name.
Please enter a letter from A to Z; If this does not apply to you please type "A"

________________________________________________________________

**End of Block: ID**

**Start of Block: Pre/Post Questions ROR_3.13.23**

Have you taken a previous version of this Reach Out and Read emotional connection training?

- Yes
- No
- Unsure

The questions below ask about your views as related to emotional connection as a construct within early relational health.

There are no right and wrong answers; we want your honest response on your perspective.

What do you think is the minimum amount of time required to observe the quality of the relationship between a parent/caregiver and an infant (in minutes)?

- I don't know
- 1
- 2
- 3
- 4
- 5
- 6
- 7
- 8
- 9
- 10
- 11
- 12
- 13
- 14
- 15
- 16
- 17
- 18
- 19
- 20
- 21
- 22
- 23
- 24
- 25
- 26
- 27
- 28
- 29
- 30
- 31
- 32
- 33
- 34
- 35
- 36
- 37
- 38
- 39
- 40
- 41
- 42
- 43
- 44
- 45
- 46
- 47
- 48
- 49
- 50
- 51
- 52
- 53
- 54
- 55
- 56
- 57
- 58
- 59
- 60
- 61
- 62
- 63
- 64
- 65
- 66
- 67
- 68
- 69
- 70
- 71
- 72
- 73
- 74
- 75
- 76
- 77
- 78
- 79
- 80
- 81
- 82
- 83
- 84
- 85
- 86
- 87
- 88
- 89
- 90
- 91
- 92
- 93
- 94
- 95
- 96
- 97
- 98
- 99
- 100
- 101
- 102
- 103
- 104
- 105
- 106
- 107
- 108
- 109
- 110
- 111
- 112
- 113
- 114
- 115
- 116
- 117
- 118
- 119
- 120

How familiar are you with the concept of emotional connection?

|  | Not familiar | Very familiar |
| --- | --- | --- |

| 2 | 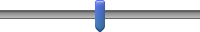 |
| --- | --- |

How confident are you in observing the quality of the parent/caregiver-infant relationship during well child visits?

|  | Not at all | Extremely |
| --- | --- | --- |

| 1 | 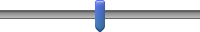 |
| --- | --- |

| Page Break |  |
| --- | --- |

Please list up to three observable elements that indicate the quality of the parent/caregiver-infant relationship.

- #1: __________________________________________________
- #2: __________________________________________________
- #3: __________________________________________________

| Page Break |  |
| --- | --- |

Please rate your agreement with the following statements

Understanding Early Relational Health is essential to my work as a clinician

|  | Strongly Disagree | Neither agree nor disagree | Strongly agree | I do not know what this is |
| --- | --- | --- | --- | --- |

| 2 | 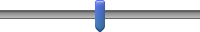 |
| --- | --- |

Observing emotional connection in well-child visits will improve my practice as a clinician.

|  | Strongly Disagree | Neither agree nor disagree | Strongly agree | I do not know what this is |
| --- | --- | --- | --- | --- |

| 3 | 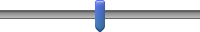 |
| --- | --- |

Observing emotional connection in well-child visits will improve outcomes for patients and their families.

|  | Strongly Disagree | Neither agree nor disagree | Strongly agree | I do not know what this is |
| --- | --- | --- | --- | --- |

| 4 | 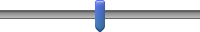 |
| --- | --- |

**End of Block: Pre/Post Questions ROR_3.13.23**

**Start of Block: Outcomes**

| 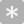 |
| --- |

What do you anticipate as primary barriers to integrating observation of emotional connection into your practice? Select up to 3 responses.

- Knowledge of how to do this
- Time required to do this
- The focus/energy required to do this
- Families may not be open to talking about this
- Cultural differences between me and my patients/families
- Language differences between me and my patients/families
- Knowing what to do if I identify a problem
- Clinic support/capacity for supporting families
- Other (specify) __________________________________________________

The following questions address your overall experience with this training. 

Please rate your level of agreement with the following statements:

|  | 1 Strongly disagree | 2 | 3 | 4 Neither agree nor disagree | 5 | 6 | 7 Strongly agree |
| --- | --- | --- | --- | --- | --- | --- | --- |
| This training gave me valuable knowledge |  |  |  |  |  |  |  |
| This online module was a good way to learn about emotional connection |  |  |  |  |  |  |  |
| The strategies presented through this training are things that I could implement in my practice |  |  |  |  |  |  |  |

| Page Break |  |
| --- | --- |

What, if anything, would you change about this training?

________________________________________________________________

________________________________________________________________

________________________________________________________________

________________________________________________________________

________________________________________________________________

How would you describe the length of the training?

- Too short
- Just about right
- Too long

Would you recommend this training for Reach Out and Read clinicians?

- Yes
- No
- Unsure

How would you recommend that this training is incorporated into residency training?

- Required for all residents
- Recommended (not required)
- Available but not promoted
- Would not recommend including in residency training
- Other (specify) __________________________________________________
- I don't know

**End of Block: Outcomes**
