# Supplementary material for: Implementation of Emotional Connection Training in Pediatric Primary Care: Mixed Methods Study
Source: JMIR Med Educ. 2026 Jun 16;12:e81250. doi: 10.2196/81250 (PMC13271710; doi:10.2196/81250)
Supplement: Multimedia Appendix 2 [file mededu-v12-e81250-s002.docx]

**Focus Group Guide**

**Introduction / Consent**

Thank you so much for speaking with me today! I’m [*name*], with Duke’s Social Science Research Institute. I will be moderating this focus group, which basically means I will be facilitating our conversation. I expect that we’ll be talking for about an hour and a half. [*Introduce second focus group administrator]*

To give you a bit of background about why we’re here: The Carolinas Collaborative is working with an independent evaluation consultant, Duke’s Social Science Research Institute (SSRI), to better understand residents’ experiences with the new Reach Out and Read (RoR) training module titled “*Incorporating Observation of Emotional Connection into Delivery of the Reach Out and Read Model.”*

As part of this research, we have invited residents who completed the module to participate in a brief focus group. Your thoughts and opinions will help to inform the development of this and other training efforts.

I want to note a few points about our conversation:

- My role as moderator will be to guide the discussion. However, you can – and should - talk directly to each other.
- We’d like to hear many different viewpoints and would like to hear from everyone. In respect for each other, I am going to ask that only one person speak at a time.
- There are no right or wrong answers, only differing points of view. Please be fully open and honest in saying what you think.
- We would also like to ask that everyone keep what is shared in the group today within the group. I hope you can be honest, even if what you say is different from what others in the group say.
- Your participation is voluntary, meaning you don’t have to respond to every question or to participate if you don’t want to. If you want to stop taking part in this session after it has begun, just raise your hand.

After talking to you, we will summarize what we learned in aggregate, with no identification of individual respondents.

Do you have any questions at this point? *[pause and answer any questions]*

So, are you comfortable participating?  *[document response]*

We would also like to audio record the focus group, so we can go back to it at a later time. The recording will be transcribed by a third party. The recording will be retained as long as the project is active and will be destroyed when the research is completed. The audio recordings and transcripts will not be shared outside of the research team. Will that be OK with everyone here? If it is NOT ok with all, we will not record, or you can excuse yourself from the group altogether. *[document response]*

Thank you so much for your time and cooperation! We truly value your input.

Introductions

- 1. Please share your name, year of residency, and which Carolinas Collaborative program you are affiliated with.

Expectations / sense of purpose

I want to start off by talking about initial understandings you had about this training.

- 1. What, if anything, did you know about the purpose of the training module prior to engaging with it?
     1. How was it introduced or presented to you? By whom?
     2. Did you think it would be useful?
     3. Were you familiar with the concepts of Emotional Connection and/or early relational health?
  2. In your own words, what was the goal of the training module?

Experience

Now, I wanted to ask a bit about how you experienced the module.

- 1. Did you complete the module as part of a group time (like a period set aside in your program for training)? Or were you asked to complete this on your own time?
     1. [if group] Did someone actively lead this?
     2. Did you think the way you did it worked well? Why / why not?
  2. Did you feel like you gave the module your full attention? Why/ why not?
     1. Were you able to fully watch each video? Why / why not?
     2. Did you complete it? Why / why not?
  3. Now let’s talk about the content of the training module, or the information that was provided.
     1. What information was the most useful to you? Why?
     2. Had you heard this information before? If so, when/where?
     3. Was any of the information less useful? If so, what? Why?
  4. Let’s talk about the format of the module, so things like its length, use of videos, etc.
     1. What aspects of the format worked well?
     2. What aspects of the format were less effective or challenging?
     3. Is an online module an effective way to present this training? Why/why not?
  5. What, if anything, would you change about the module?

Outcomes

- 1. How, if at all, has this training benefited you?
  2. Do you think you learned more about Emotional Connection?
     1. Why / why not?
     2. [if learned anything] what did you learn?
  3. Is observation of Emotional Connection something that you think you could incorporate into your well child visits? Why/why not?
     1. What do you anticipate as potential barriers to doing this?
     2. Is there anything from the modules that was especially helpful for preparing you to incorporate this?
  4. How, if at all, do you think observing Emotional Connection during well child visits might benefit the children and/or families you serve?

Closing

- 1. Is there anything else you’d like to share?
