# Supplementary material for: Implementation of Emotional Connection Training in Pediatric Primary Care: Mixed Methods Study
Source: JMIR Med Educ. 2026 Jun 16;12:e81250. doi: 10.2196/81250 (PMC13271710; doi:10.2196/81250)
Supplement: Multimedia Appendix 3 [file mededu-v12-e81250-s003.docx]

Matrix Structure for Qualitative Rapid Analysis

| **Expectations** | **Data Collection Event #1** | **Data Collection Event #2** | **…[added data collection events with added columns]** |
| --- | --- | --- | --- |
| Understood purpose of training |  |  |  |
| How was training introduced |  |  |  |
| Expected usefulness |  |  |  |
| Prior familiarity with concepts of emotional connection |  |  |  |
| Goal of training |  |  |  |
| **Experience** |  |  |  |
| Access to training (how offered by program) |  |  |  |
| Mode of completion |  |  |  |
| Attention paid to training |  |  |  |
| Information (content) in training |  |  |  |
| Format |  |  |  |
| **Outcomes** |  |  |  |
| Emotional connection learnings |  |  |  |
| Benefits of training for clinicians |  |  |  |
| Benefits of training for families |  |  |  |
| Incorporating in pediatric training |  |  |  |
| Limiting factors to implementation |  |  |  |
| **Suggestions** |  |  |  |
| Format |  |  |  |
| Content |  |  |  |
| Logistic/technical |  |  |  |
| Changes |  |  |  |
| Added notes/Reflections |  |  |  |
| Misc/Other |  |  |  |
